# Resistance Training Data Analysis on Blood and Personal Profiles for Customized Healthcare

**Published:** 2018-07

**Authors:** Wi-Young SO, Taikyeong Ted JEONG

**Affiliations:** 1. Sports and Health Care Major, College of Humanities and Arts, Korea National University of Transportation, Chungju-si 27469, Korea; 2. Dept. of Business Intelligence, CHA University, Pocheon, Gyeonggi-do, Korea

## Dear Editor-in-Chief

Regular resistance training results in consistent improvements in muscle mass, fiber type, glycemic control, and muscle mitochondrial function (1–3). Resistance training also leads to an improvement in disease conditions, such as Type 2 diabetes (4).

College students have the best physical and psychological condition compared to other stages in their life. However, approximately 600,000 students in a healthy nation such as the United States in 2009 have reported some form of disability or medical condition such as attention-deficit/hyperactivity disorder, learning disabilities, psychiatric disorders, or chronic illnesses (5, 6). Few studies have investigated the effectiveness of resistance training on blood profiles, which indicate the physical condition, in college students. Therefore, this study aimed to examine the effects of resistance training on blood profiles in Korean college students.

Fifteen college students who visited the Exercise Physiology Laboratory in Korea National University of Transportation, Republic of Korea, were included in 2014. Informed consent to participate was obtained from all participants.

The participants underwent resistance training according to the protocol modified (7), for 60 min and 3 times per week for 12 wk. The participants underwent blood profile analysis before and after the intervention. A specialist nurse obtained the blood samples from the participant’s forearm vein (10 mL) in the morning after a 12-h fast, using a vacuum blood-gathering tube. The blood profile was obtained using the ADVIA 1650 automated analyzer (Bayer HealthCare Ltd. Tarrytown, NY, USA). All data are presented as mean ± standard deviation and analyzed using paired *t*-test. All analyses were performed using SPSS (ver. 18.0, Chicago, IL, USA). Statistical significance was set at *P*<0.05.

The participant characteristics were as follows: male, n = 11, female, n = 4; age, 21.20 ± 1.26 yr; height, 171.20 ± 5.72 cm; weight, 64.13 ± 11.01 kg; and body mass index, 21.76 ± 2.85 kg/m^2^. Blood profile analysis showed that there were no significant differences between pre and post-intervention for red blood cell count (*P*=0.580), white blood cell count (*P*=0.676), hematocrit (*P*=0.207), blood platelets (*P*=0.170), and fasting glucose (*P*=0.280), total cholesterol (*P*=0.287), high-density lipoprotein (*P*=0.594), low-density lipoprotein (*P*=0.419), and triglyceride (*P*=0.092) levels (Table 1).

**Table 1:** Changes in blood profiles after 12 wk of resistance exercise

***Variables***	***Before***	***After***	***t***	***P***
Red blood cell (count/mm^3^)	4.86 ± 0.47	4.91 ± 0.44	−0.567	0.580
White blood cell (count/mm^3^)	6.71 ± 1.39	6.55 ± 1.51	0.427	0.676
Hematocrit (%)	44.96 ± 5.17	43.89 ± 3.82	1.322	0.207
Blood platelets (count/mm^3^)	243.20 ± 41.69	254.93 ± 49.22	−1.446	0.170
Fasting glucose (mg/dl)	88.93 ± 10.27	92.73 ± 11.65	−1.123	0.280
Total cholesterol (mg/dl)	181.27 ± 22.15	176.33 ± 24.41	1.107	0.287
High-density lipoprotein (mg/dl)	65.47 ± 14.44	67.00 ± 13.57	−0.545	0.594
Low-density lipoprotein (mg/dl)	101.79 ± 22.34	106.27 ± 22.55	−0.833	0.419
Triglyceride (mg/dl)	94.00 ± 28.97	78.20 ± 31.53	1.808	0.092

Data are presented as mean ± standard deviation

Tested by paired *t*-test

Twelve-week resistance exercise did not affect blood profiles in a sample of Korean college students. Therefore, other types of exercises such as aerobic exercises and not resistance training may be recommended to improve blood profiles in young students.
